# Telotristat ethyl, a tryptophan hydroxylase inhibitor, enhances antitumor efficacy of standard chemotherapy in preclinical cholangiocarcinoma models

**DOI:** 10.1111/jcmm.18585

**Published:** 2024-09-02

**Authors:** Niranjan Awasthi, Lily Darman, Margaret A. Schwarz, Roderich E. Schwarz

**Affiliations:** ^1^ Department of Surgery Indiana University School of Medicine South Bend Indiana USA; ^2^ Harper Cancer Research Institute University of Notre Dame Notre Dame Indiana USA; ^3^ Department of Chemistry and Biochemistry University of Notre Dame Notre Dame Indiana USA; ^4^ Department of Pediatrics Indiana University School of Medicine South Bend Indiana USA; ^5^ Roswell Park Comprehensive Cancer Center Buffalo New York USA

**Keywords:** cholangiocarcinoma, gemcitabine, *nab*‐paclitaxel, serotonin, telotristat

## Abstract

Cholangiocarcinoma (CCA), an aggressive biliary tract cancer, carries a grim prognosis with a 5‐year survival rate of 5%–15%. Standard chemotherapy regimens for CCA, gemcitabine plus cisplatin (GemCis) or its recently approved combination with durvalumab demonstrate dismal clinical activity, yielding a median survival of 12–14 months. Increased serotonin accumulation and secretion have been implicated in the oncogenic activity of CCA. This study investigated the therapeutic efficacy of telotristat ethyl (TE), a tryptophan hydroxylase inhibitor blocking serotonin biosynthesis, in combination with standard chemotherapies in preclinical CCA models. Nab‐paclitaxel (NPT) significantly enhanced animal survival (60%), surpassing the marginal effects of TE (11%) or GemCis (9%) in peritoneal dissemination xenografts. Combining TE with GemCis (26%) or NPT (68%) further increased survival rates. In intrahepatic (iCCA), distal (dCCA) and perihilar (pCCA) subcutaneous xenografts, TE exhibited substantial tumour growth inhibition (41%–53%) compared to NPT (56%–69%) or GemCis (37%–58%). The combination of TE with chemotherapy demonstrated enhanced tumour growth inhibition in all three cell‐derived xenografts (67%–90%). PDX studies revealed TE's marked inhibition of tumour growth (40%–73%) compared to GemCis (80%–86%) or NPT (57%–76%). Again, combining TE with chemotherapy exhibited an additive effect. Tumour cell proliferation reduction aligned with tumour growth inhibition in all CDX and PDX tumours. Furthermore, TE treatment consistently decreased serotonin levels in all tumours under all therapeutic conditions. This investigation decisively demonstrated the antitumor efficacy of TE across a spectrum of CCA preclinical models, suggesting that combination therapies involving TE, particularly for patients exhibiting serotonin overexpression, hold the promise of improving clinical CCA therapy.

## 1 INTRODUCTION

Cholangiocarcinoma (CCA) is a highly aggressive biliary tract cancer, ranking as the second most prevalent primary hepatobiliary malignancy following hepatocellular carcinoma. It accounts for approximately 15% of primary liver cancers and 3% of all gastrointestinal (GI) cancers.^1^ CCA is categorized based on its anatomical site of origin into three main types: intrahepatic CCA (iCCA), perihilar CCA (pCCA) and distal CCA (dCCA).^2^ iCCA originates in or above the second‐order bile ducts, while the point of anatomical differentiation between pCCA and dCCA is the transition from the common hepatic duct into the common bile duct at the insertion of the cystic duct. pCCA and dCCA can also be collectively referred to as extrahepatic (eCCA).^3^ In the United States, pCCA represents the largest subgroup, accounting for approximately 50%–60% of all CCAs, followed by dCCA (20%–30%) and iCCA (10%–20%).^4^, ^5^, ^6^ A recent retrospective analysis demonstrated that CCA incidence continued to increase (43.8%) from 2001 to 2017 in the US, with a greater increase in iCCA (148.8%) compared to eCCA (7.5%).^7^

Over the past decades, the incidence and mortality rates of CCA have been steadily rising worldwide, constituting a global health challenge.^6^, ^8^ This cancer is associated with a particularly grim prognosis, primarily due to late‐stage diagnosis, resulting in a 5‐year survival rate ranging from 5% to 15%.^9^ Surgical resection remains the sole curative treatment option for CCA. However, only a minority of patients, approximately 35%, present with early‐stage disease amenable to curative intent resection.^10^ In cases where surgical intervention is not feasible, the standard cytotoxic chemotherapy regimen, gemcitabine plus cisplatin (GemCis), is employed, despite offering limited clinical benefit, as evidenced by a median survival of just 14 months.^11^ Recently, durvalumab, an antibody targeting the immunosuppressive programmed death‐ligand 1 (PD‐L1) protein, received FDA approval for CCA patients when used in combination with GemCis. This approval was based on significantly improved clinical results compared to a placebo plus GemCis combination, including extended overall survival (OS, 12.8 vs. 11.5 months) and progression‐free survival (PFS, 7.2 vs. 5.7 months).^12^ Given the high toxicity and limited efficacy of current treatments, there is a pressing need for novel and more effective therapeutic strategies to improve the prospects of CCA patients.

Nanoparticle formulations of conventional chemotherapies have exhibited several advantages, including an improved safety profile, enhanced drug distribution, increased tumour penetration and prolonged retention. These factors contribute to heightened cytotoxic responses in both epithelial tumour cells and the stromal microenvironment.^13^ *Nab*‐paclitaxel (NPT), an albumin‐bound formulation of paclitaxel, has gained approval as a therapy for breast cancer, non‐small cell lung cancer (NSCLC), and pancreatic cancer.^14^ It is currently under clinical investigation for several solid tumours, including CCA, based on its demonstrated superior antitumor efficacy and safety profile.^15^

Serotonin (5‐hydroxytryptamine, 5‐HT) is a biogenic monoamine synthesized in enterochromaffin cells throughout the gastrointestinal tract and in serotonergic neurons in the central nervous system (CNS).^16^, ^17^ It is produced through a systematic process involving hydroxylation and decarboxylation of the essential amino acid tryptophan, catalysed by the enzymes tryptophan hydroxylase (TPH) and amino acid decarboxylase.^16^ TPH serves as the rate‐limiting enzyme in serotonin synthesis, with two isoforms, TPH1 and TPH2. TPH2 synthesizes serotonin in the brain, while TPH1 is responsible for serotonin synthesis in the gut and other peripheral tissues.^18^ Approximately 95% of total body serotonin is produced in peripheral organs, with the remaining 5% originating in the brain.^19^ In addition to its role as a brain neurotransmitter in the CNS, serotonin plays multiple extra‐neuronal physiological and pathophysiological roles.^20^ Serotonin has been implicated in the autocrine loops of growth factors, contributing to cell proliferation in several aggressive cancers, including small cell lung carcinoma, prostate cancer, breast cancer and bladder cancer.^21^, ^22^, ^23^, ^24^ In a pancreatic ductal adenocarcinoma (PDAC) study, elevated levels of TPH1 and decreased levels of monoamine oxidase A (MAOA), a serotonin‐degrading enzyme, were reported, correlating with tumour stage, size and poor prognosis. In this study, a serotonin receptor 5‐HT2B antagonist slowed tumour growth and extended the survival of mice in xenograft studies.^25^ Notably, in CCA patient samples, Alpini et al. reported an increased accumulation and secretion of serotonin. Their study also revealed the stimulatory effects of serotonin on CCA cell growth, and the specific inhibition of serotonin production resulted in tumour growth inhibition in CCA xenografts.^26^ These findings underscore the dysregulation of serotonin metabolism in CCA and highlight the therapeutic potential of serotonin inhibition in this disease.

A promising approach to inhibit peripheral serotonin synthesis is targeting the enzyme TPH1 with telotristat ethyl (TE, Figure S1). TE, a prodrug of telotristat, serves as a novel TPH1 inhibitor with high‐molecular‐weight and acidic moieties, preventing it from crossing the blood–brain barrier. Consequently, TE is specifically designed to inhibit peripheral serotonin production without affecting serotonin levels in the CNS.^27^ TE is already an FDA‐approved treatment for carcinoid syndrome diarrhoea.^28^

Given the increased serotonin levels observed in CCA, along with the known oncogenic potential of serotonin in several cancers, it is reasonable to speculate that serotonin may exhibit pro‐tumorigenic activity in CCA, potentially offering a target for therapeutic interventions. This study is the first to demonstrate the antitumor efficacy of a serotonin biosynthesis inhibitor, TE, across different CCA subtypes using cell‐derived xenografts (CDX) and patient‐derived xenografts (PDX). Additionally, our findings reveal TE's capacity to enhance the therapeutic response of standard cytotoxic regimens GemCis and nanoparticle paclitaxel, in preclinical models. The results of this study suggest that TE holds promise for improving clinical outcomes in CCA, particularly in patients with serotonin overexpression.

## 2 MATERIALS AND METHODS

### 2.1 Cell culture and reagents

Human iCCA CC‐LP‐1 cells, dCCA TFK‐1 cells and pCCA SNU‐1196 cells were cultured in DMEM or RPMI medium (Sigma Chemical Co., St. Louis, MO) supplemented with 10% fetal bovine serum (FBS). Cell cultures were maintained at 37°C in a humidified incubator with 5% CO_2_ and 95% air. Detailed characteristics of these CCA cell lines are provided in Table S1. Therapeutic agents gemcitabine, cisplatin and *nab*‐paclitaxel, were procured from the pharmacy at the Goshen Center for Cancer Care (Goshen, IN). Telotristat ethyl was obtained from TerSera Therapeutics (Deerfield, IL).

### 2.2 Western blot analysis

Subconfluent monolayers of CCA cells were treated with gemcitabine, cisplatin, *nab‐*paclitaxel, and TE, followed by a 16‐h incubation. Whole‐cell lysates were prepared. Tumour lysates were prepared using tumour tissues obtained from subcutaneous xenografts that were snap‐frozen and stored at −80°C. These tissues were suspended in lysis buffer containing protease and phosphatase inhibitors and homogenized in a Bullet Blender Homogenizer (Next Generation, Averill Park, NY). Extracts were centrifuged and the supernatant lysate was collected. The protein concentration was measured using a Bradford assay. Proteins were separated on 10% polyacrylamide gels via electrophoresis and transferred to a polyvinylidene fluoride (PVDF) membrane (Bio‐Rad, Hercules, CA) for immobilization. The membranes were incubated at 4°C overnight with specific primary antibodies, followed by a 1‐2‐h incubation with the corresponding horseradish peroxidase (HRP)‐conjugated secondary antibodies (Cell Signalling Technology, Danvers, MA). Protein bands were visualized using an enhanced chemiluminescence reagent in an Image360 system, and densitometry analysis was conducted. The intensity of the protein bands was quantified using ImageJ software. Mean densitometric values of independent protein bands were normalized to the mean densitometric values of their respective total protein or glyceraldehyde‐3‐phosphate dehydrogenase (GAPDH) and presented as relative protein expression levels.

### 2.3 Animal studies

All animals were housed in a pathogen‐free facility with ad libitum access to food and water, and their care was in accordance with institutional guidelines. Animal experiments were conducted following a protocol approved by the Institutional Animal Care and Use Committee (IACUC) at the Indiana University School of Medicine (South Bend, IN). Female nonobese diabetic/severe combined immunodeficient (NOD/SCID) mice (4–6 weeks of age) were purchased from Charles River Laboratories (Wilmington, MA).

#### 2.3.1 Animal survival study

Animal survival studies were performed in peritoneal dissemination xenografts using NOD/SCID mice. The mice were intraperitoneally injected with 10 × 10^6^ CC‐LP‐1 cells per mouse. Ten days post‐tumour cell injection, all mice were randomized (*n* = 6–7 per group) to receive phosphate‐buffered saline (control), gemcitabine (50 mg/kg, twice a week), cisplatin (2.5 mg/kg, twice a week), *nab*‐paclitaxel (5 mg/kg, twice a week), or TE (100 mg/kg, 5 days per week) for 2 weeks. Animals were evaluated daily for any drug‐related toxicities. After completing treatment, mice were monitored daily and euthanized when moribund based on predefined criteria, including sudden weight loss or gain (>15%), lethargy, inability to remain upright, and lack of strength.^29^ Animal survival was evaluated from the first day of treatment until death.

#### 2.3.2 Tumour growth inhibition studies in CDX models

Human CCA CC‐LP‐1 or TFK‐1 cells (10 × 10^6^) were subcutaneously implanted into the right flank region of NOD/SCID mice. Ten days post‐tumour cell injection, all mice had measurable tumours. Mice were then randomized to ensure at least five mice per group. In the CC‐LP‐1 xenograft experiment, mice were treated with PBS (control), gemcitabine (50 mg/kg, twice a week), cisplatin (2.5 mg/kg, twice a week), *nab*‐paclitaxel (5 mg/kg, twice a week), or TE (100 mg/kg, 5 days per week), for 2 weeks. In the TFK‐1 or SNU‐1196 xenograft experiments, mice were treated with PBS (control), gemcitabine (25 mg/kg, twice a week), cisplatin (1.25 mg/kg, twice a week), *nab*‐paclitaxel (5 mg/kg, twice a week) or TE (100 mg/kg, 5 days per week), for 2 weeks. Tumour size was measured twice per week, and tumour volume was calculated using the formula *V* = 1/2 (*L* × *W* ^2^), where *L* = length and *W* = width. After the completion of treatment, mice were euthanized, and tumours were dissected and processed for histological, immunohistochemical, and Western blot analysis.

#### 2.3.3 Tumour growth inhibition studies in PDX models

NOD‐SCID gamma (NSG) mice carrying CCA patient‐derived tumours (TM00311 and TM01225) were purchased from the Jackson Laboratory (Bar Harbour, ME). Detailed characteristics of these PDX tumours are provided in Table S2. The tumour was sectioned into small pieces (1–2 mm^3^) and subcutaneously implanted into the right flanks of NSG mice under anaesthesia with isoflurane. Tumour growth was monitored twice weekly using a calliper. When the tumour volume reached approximately 1000 mm^3^, tumours were harvested for serial transplantation into NSG mice and used for the experiment when tumour volume reached about 100–200 mm^3^. PDX‐bearing mice were randomized, grouped, and treated for 2 weeks as mentioned previously in the CDX model study. The tumour size was measured, and tumour volume was calculated.

### 2.4 Immunohistochemistry (IHC) and immunofluorescence (IF) analysis

Subcutaneous tumours were fixed in 4% paraformaldehyde, dehydrated through a graded ethanol series (25%–100%), embedded in paraffin, and sectioned. Tumour sections (5 μm) were deparaffinized and rehydrated, and heat‐mediated antigen retrieval was performed in citrate buffer. Subsequently, tumour sections were blocked with CAS buffer for 20 min.

Tumour cell proliferation was assessed by incubating the sections overnight at 4°C with an anti‐Ki67 antibody (Abcam, Cambridge, MA), followed by a 40‐min incubation at room temperature with a Cy3 secondary antibody. Slides were mounted using a fluorescence mounting solution, and images were captured under a fluorescence microscope.

Serotonin expression was determined by incubating the sections overnight at 4°C with an anti‐serotonin antibody (Novus Biologicals; NB100‐65037), followed by a 40‐min incubation with a Cy3 secondary antibody at room temperature. Slides were mounted using a fluorescence mounting solution, and images were acquired under a fluorescence microscope. Fluorescence microscopy was performed using the Olympus microscope IX81, and images were obtained with a Hamamatsu Orca digital camera (Hamamatsu Corporation, Bridgewater, NJ) equipped with a DSU spinning confocal unit and analysed using cellSens Dimension software (Olympus, Center Valley, PA). Tumour cell proliferation was analysed by counting Ki67‐positive cells from 5 high‐power fields (HPF), while serotonin expression was analysed by measuring fluorescence intensity using ImageJ software.

### 2.5 Statistical analysis

The two‐tailed Student's *t*‐test (GraphPad Prism 7.0 Software, San Diego, CA) was utilized to analyse the statistical significance for individual group comparisons. For in vivo tumour growth studies, statistical analysis was conducted using one‐way ANOVA for multiple group comparisons and the Student's *t*‐test for individual group comparisons. Nonparametric testing with log‐rank group comparisons (GraphPad Prism 7.0) was applied for survival study statistics. Statistical significance was determined based on the *p*‐value between control and therapy groups and marked accordingly (not significant: *p* > 0.05; *p < 0.05; **p < 0.01; ***p < 0.001; ****p < 0.0001), between TE and combination therapy groups (not significant: *p* > 0.05; •*p* < 0.05; ••*p* < 0.01; •••*p* < 0.001; •••*p* < 0.0001), and between chemotherapy and combination therapy groups (not significant: *p* > 0.05; #*p* < 0.05; ##*p* < 0.01; ###*p* < 0.001; ####*p* < 0.0001). In animal experiments, G*Power 3.1 software was used for the power calculations. A sample size of six to eight mice per group was employed in animal survival experiments, while five mice per group were used in subcutaneous tumour growth experiments. With a sample size of five to eight mice per group, a preset *α* value of 0.05, statistically significant differences in animal survival or tumour size of 40%, and a standard deviation of 20%, the experiments could detect significant effects with a power of greater than 80%.

## 3 RESULTS

### 3.1 Animal survival benefits of telotristat ethyl and cytotoxic agents

Considering that the peritoneum is the most common site of metastatic disease in CCA patient, we assessed the efficacy of TE and cytotoxic drugs in improving animal survival using a peritoneal dissemination model with iCCA CC‐LP‐1 cells. After 2 weeks of therapy with TE and cytotoxic agents, the median animal survival in controls was 47 days. Treatment with TE alone resulted in a slight increase in animal survival (52 days), while GemCis exhibited a modest increase (51 days), which was not statistically significant compared to controls. However, *nab*‐paclitaxel resulted in a significant improvement in animal survival (75 days), representing a 59% increase (*p* = 0.0004).

**FIGURE 1:**
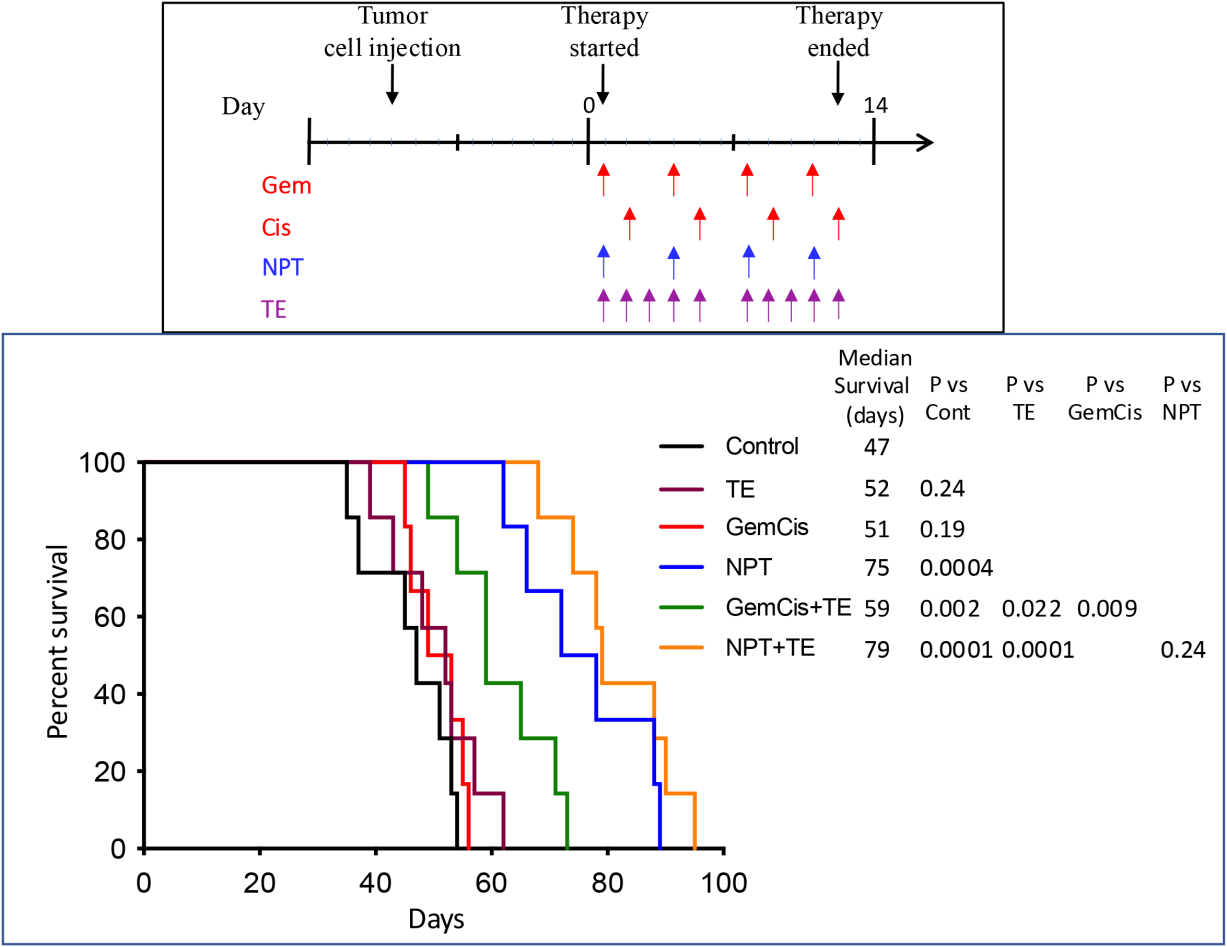
Animal survival benefits of TE and cytotoxic regimens: In CC‐LP‐1 (*n* = 7) cell‐derived peritoneal dissemination xenografts, 10 days after tumour cell injection, mice were treated with TE, GemCis, NPT or their combinations for 2 weeks. The curve represents the animal survival time from the start of therapy. Statistical group differences in survival time were calculated using log‐rank testing.

Notably, the combination of TE with chemotherapy, either GemCis or NPT, demonstrated a substantial increase in animal survival: GemCis+TE (59 days, 26% increase, *p* = 0.002) and NPT + TE (79 days, a 68% increase, *p* = 0.0001). These results were significantly higher than the survival rates observed with TE or GemCis alone (Figure 1). No significant changes in body weight were observed in mice treated with TE, NPT or NPT + TE. However, in the GemCis and GemCis+TE groups, mouse weight loss was observed during the second week of therapy, which led to the discontinuation of the last dose of gemcitabine and cisplatin.

### 3.2 Tumour growth inhibition by telotristat ethyl and cytotoxic agents in cell‐derived xenografts

**FIGURE 2:**
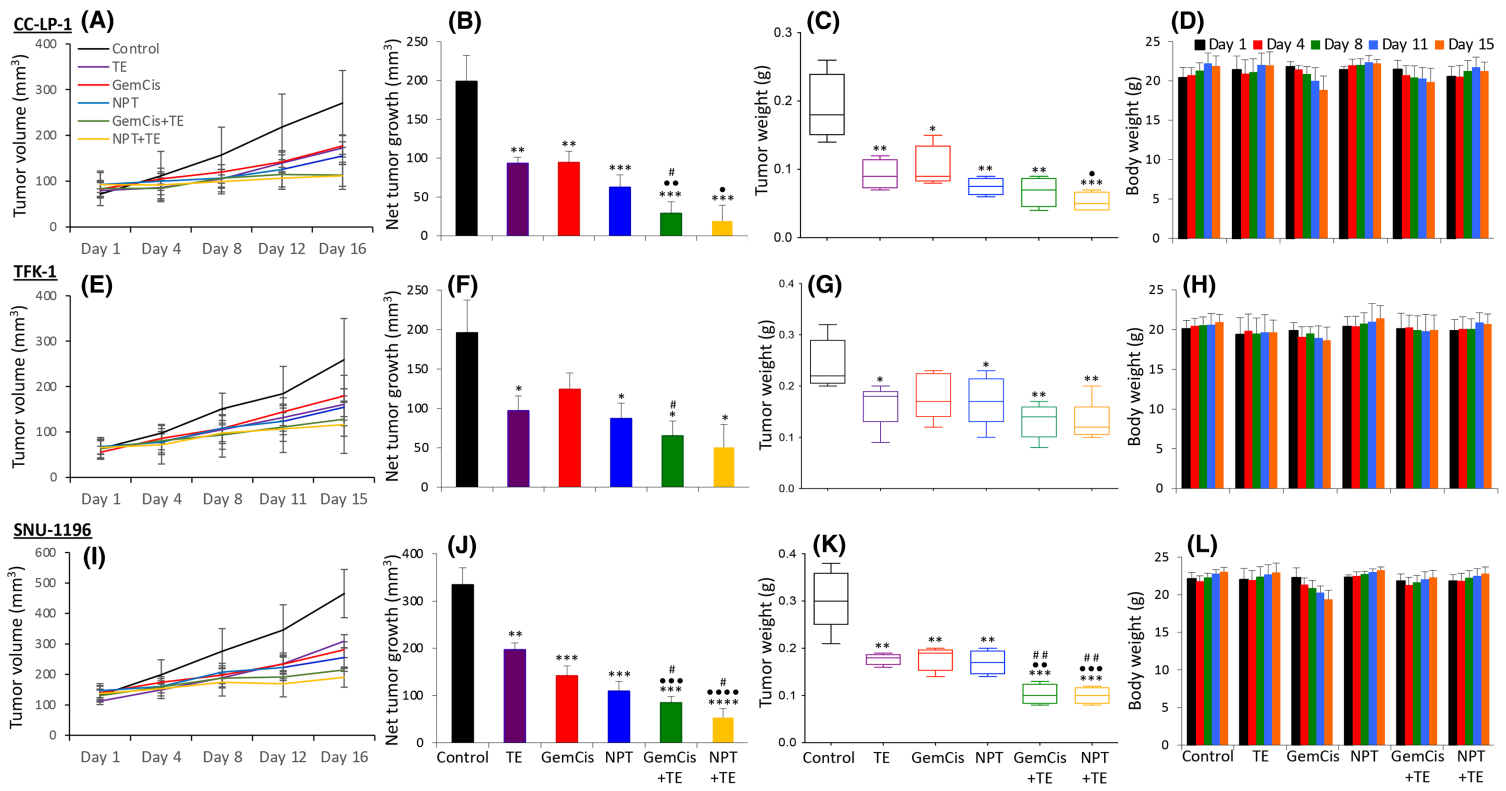
Reduction in tumour growth by TE, GemCis and NPT in CCA cell‐derived xenografts: iCCA CC‐LP‐1, dCCA TFK‐1 or pCCA SNU‐1196 cells were implanted subcutaneously in the right flank of NOD‐SCID mice. Ten days after tumour cell injection, when all mice had measurable tumours, they were treated with TE, GemCis, NPT or their combinations for 2 weeks. (A, E, I) Tumour size was measured twice a week during the therapy period using callipers and plotted. (B, F, J) Net growth in tumour size was calculated by subtracting tumour volume on the first treatment day from that on the final day and plotted as a bar graph. (C, G, K) On the final therapy day, tumours were excised, weighed, and mean tumour weight was calculated and presented as a Box plot. (D, H, L) Animal weight was measured twice a week during the therapy period and plotted. Data are representative of mean values ± standard deviation from at least five mice per group. Student's *t*‐test was performed between control and therapy groups (*p < 0.05; **p < 0.01; ***p < 0.001; ****p < 0.0001), between TE and combination therapy groups (•*p* < 0.05; ••*p* < 0.01; •••*p* < 0.001; ••••*p* < 0.0001), and between chemotherapy and combination therapy groups (#*p* < 0.05; ##*p* < 0.01).

In iCCA CC‐LP‐1 xenografts, treatment with TE, GemCis, or NPT resulted in significant inhibition of tumour growth. Importantly, the combination of TE with cytotoxic agents demonstrated a synergistic effect (Figure 2A). Net tumour growth, compared to controls (199 mm^3^), was reduced to 93 mm^3^ (TE), 94 mm^3^ (GemCis), 62 mm^3^ (NPT), 29 mm^3^ (GemCis+TE), and 19 mm^3^ (NPT+TE) (Figure 2B). Mean tumour weight at sacrifice correlated with the tumour growth inhibition, decreasing from the control group's 0.187 g to the range of 0.07–0.1 g with TE or cytotoxic therapy. Notably, combination therapy exhibited the most significant reduction in tumour weight: 0.065 g (GemCis+TE), and 0.051 g (NPT+TE) (Figure 2C). TE, NPT or NPT + TE therapy did not result in significant changes in the body weight of mice, and no treatment‐related toxicities were noted (Figure 2D). Similar to the survival experiment, some weight loss was observed in the GemCis therapy groups, leading to the discontinuation of the last dose of GemCis. Importantly, the weight loss with GemCis+TE was less than that with GemCis therapy.

In dCCA TFK‐1 xenograft study, gemcitabine and cisplatin doses were reduced based on CC‐LP‐1 xenograft experiments. Compared to CC‐LP‐1 xenografts, TE exhibited a similar effect on tumour growth inhibition, while the effects of GemCis or NPT were less pronounced. Net tumour growth, compared to controls (196 mm^3^), was reduced to 97 mm^3^ (TE), 124 mm^3^ (GemCis), and 87 mm^3^ (NPT). It was further reduced to 65 mm^3^ (GemCis+TE) and 50 mm^3^ (NPT + TE) (Figure 2F). Mean tumour weight correlated with the tumour growth inhibition, with the control group's mean tumour weight of 0.24 g reduced to 0.16 g (TE), 0.18 g (GemCis), and 0.17 g (NPT). The tumour weight was further reduced to 0.13 g (GemCis+TE) and 0.13 g (NPT+TE) (Figure 2G). Similar to CC‐LP‐1 xenografts, there was no significant change in the body weight of mice with TE, NPT or NPT+TE therapy. However, in this experiment too, weight reduction was observed with GemCis therapy, but the addition of TE mitigated this (Figure 2H).

In pCCA SNU‐1196 xenografts, TE, GemCis, and NPT inhibited tumour growth, and the addition of TE to chemotherapy regimens showed a trend for synergistic effects (Figure 2I). Net tumour growth was 334 mm^3^ in controls, reduced to 197 mm^3^ (TE), 142 mm^3^ (GemCis), and 109 mm^3^ (NPT). After combination therapy, further reduction occurred to 84 mm^3^ (GemCis+TE) and 53 mm^3^ (NPT+TE), respectively (Figure 2J). The mean tumour weight was highest in the control group (0.3 g), decreased with TE (0.18 g), GemCis (0.18 g), or NPT (0.17 g). The addition of TE to GemCis or NPT further decreased the tumour weight: GemCis plus TE (0.1 g) and NPT plus TE (0.09 g) (Figure 2K). There was no therapy‐related change in the body weight of mice with TE, NPT and NPT + TE therapy. Once again, there was some body weight loss with GemCis therapy, but this was not observed after the addition of TE (Figure 2L).

### 3.3 Tumour growth inhibition by telotristat ethyl and cytotoxic agents in patient‐derived xenografts

**FIGURE 3:**
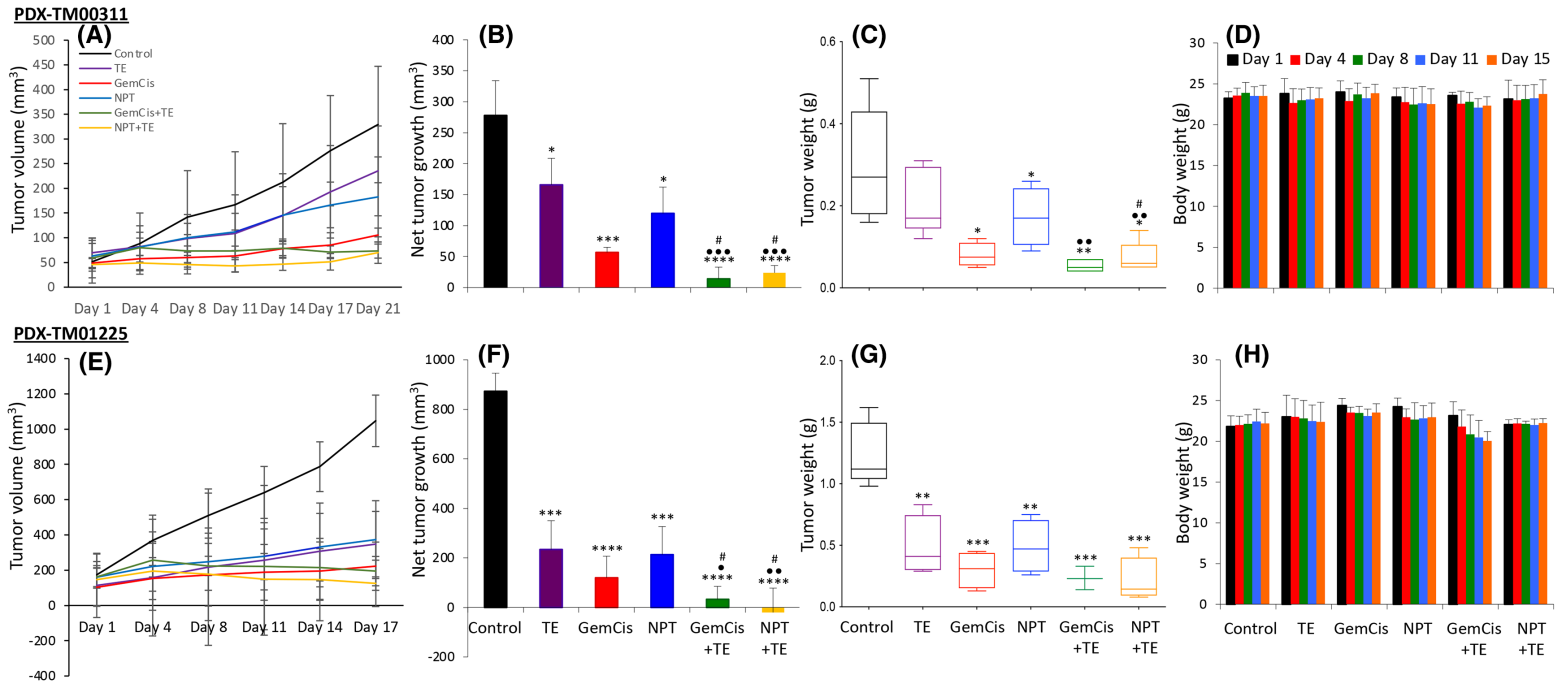
Reduction in tumour growth by TE, GemCis and NPT in CCA patient‐ derived xenografts: PDX TM00311 or TM01225 tumours were sectioned, and small pieces (1 mm × 1 mm) were implanted subcutaneously in the right flank of NSG mice. When all mice had measurable tumours, they were treated with TE, GemCis, NPT or their combinations for 2–3 weeks. (A, E) Tumour size was measured twice a week during the therapy period using callipers and plotted. (B, F) Net growth in tumour size was calculated by subtracting tumour volume on the first treatment day from that on the final day and plotted as a bar graph. (C, G) On the final therapy day, tumours were excised, weighed, and mean tumour weight was calculated and presented as a Box plot. (D, H) Animal weight was measured twice a week during the therapy period and plotted. Data are representative of mean values ± standard deviation from at least five mice per group. Student's *t*‐test was performed between control and therapy groups (*p < 0.05; **p < 0.01; ***p < 0.001; ****p < 0.0001), between TE and combination therapy groups (•*p* < 0.05; ••*p* < 0.01; •••*p* < 0.001), and between chemotherapy and combination therapy groups (#*p* < 0.05).

In a PDX TM00311 study, TE, GemCis, NPT or their combinations exhibited significant delays in tumour growth (Figure 3A). Unlike the CDX studies, GemCis exhibited a more pronounced tumour inhibition effect than NPT. The net increase in tumour size in the control group was 278 mm^3^, which decreased to 166 mm^3^ with TE, 57 mm^3^ with GemCis, and 120 mm^3^ with NPT treatment. A synergistic reduction in tumour growth was observed in the combination therapy groups GemCis+TE (14 mm^3^) and NPT + TE (24 mm^3^) (Figure 3B). The mean tumour weight was greatest in the control group (0.27 g), decreasing with TE (0.19 g), GemCis (0.08 g) or NPT (0.16 g) therapy. The addition of TE to the chemotherapy regimens exhibited a synergistic response (Figure 3C). Therapies appeared to be well tolerated, with no significant changes in mouse weight observed (Figure 3D).

In another PDX TM01225 study, tumour growth was considerably faster than TM00311. TE, GemCis, or NPT delayed tumour growth, and once again, GemCis had a more pronounced tumour inhibition effect than NPT, and the addition of TE with chemotherapy regimens had a synergistic effect (Figure 3E). The net increase in tumour size in the control group was 873 mm^3^, which decreased to 234 mm^3^ with TE, 120 mm^3^ with GemCis, 213 mm^3^ with NPT treatment. The combination therapy with TE caused a further reduction in net tumour growth: GemCis+TE (33 mm^3^) and NPT + TE (−20 mm^3^, indicating tumour regression) (Figure 3F). The mean tumour weight was highest in the control group (1.2 g), decreasing with TE (0.44 g), GemCis (0.27 g), or NPT (0.45 g) therapy. The addition of TE to chemotherapy exhibited a synergistic response: GemCis plus TE (0.22 g) and NPT plus TE (0.17 g) (Figure 3G). In this study too, therapies appeared to be well tolerated, with no significant changes in the body weight of mice (Figure 3H).

### 3.4 Telotristat ethyl and cytotoxic agents' effect on intratumor proliferation

**FIGURE 4:**
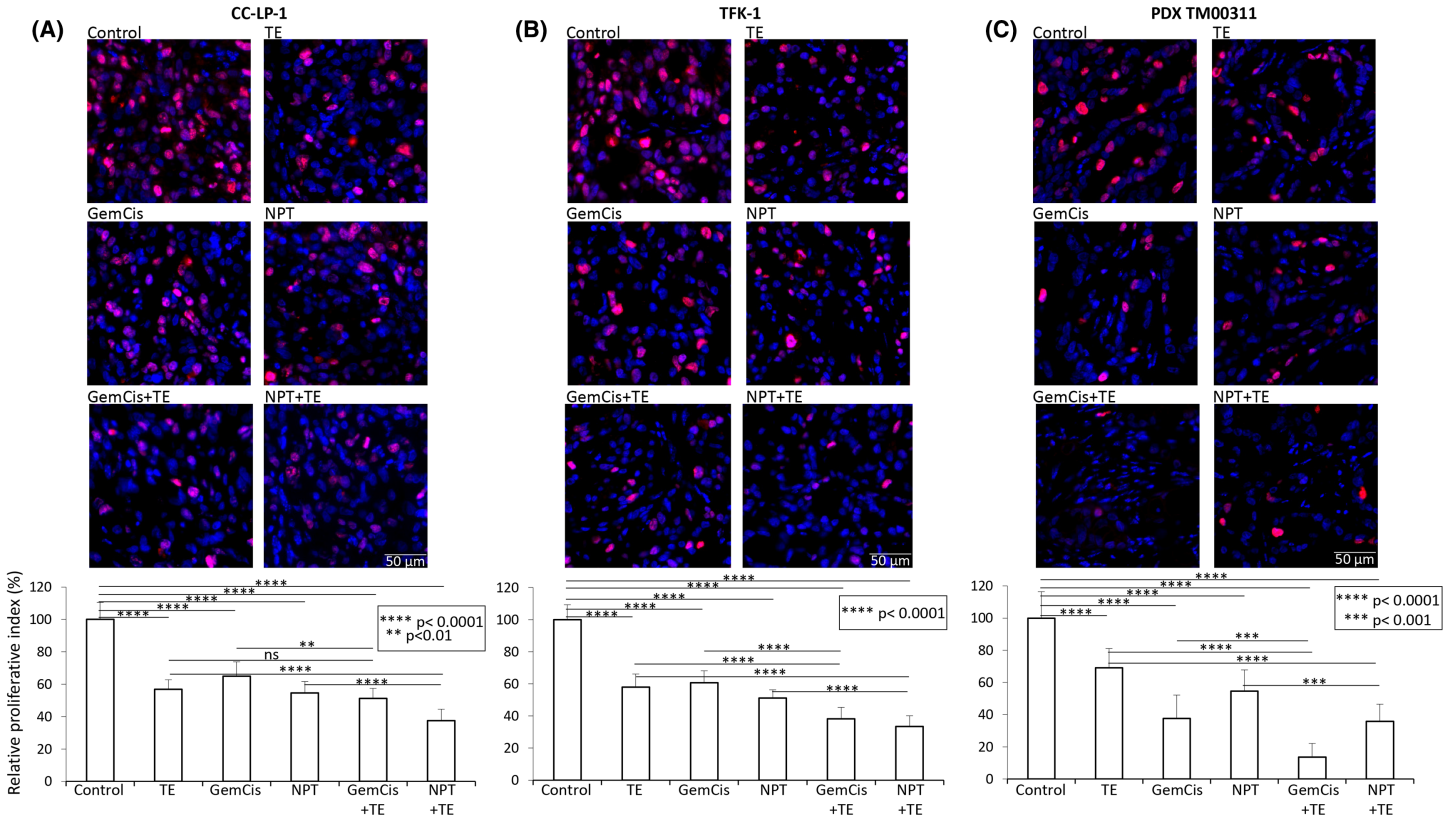
Reduction in tumour cell proliferation by TE, GemCis and NPT treatment in CCA tumours: Tumour sections from (A) iCCA CC‐LP‐1, (B) dCCA TFK‐1, and (C) PDX TM00311 subcutaneous xenografts after 2 weeks of treatment were immunostained with Ki67 antibody and photographed under a fluorescent microscope. Ki67‐positive cells were counted in five different high‐power fields (HPF). The upper panel depicts merged images of cell nuclei stained with Ki67 (red) and DAPI (blue) illustrated at 20× magnification. The lower panel bar chart depicts the mean ± standard deviation. Student's *t*‐test was performed between control and therapy groups, between TE and combination therapy groups, and between chemotherapy and combination therapy groups (**p < 0.01; ***p < 0.001; ****p < 0.0001).

To investigate the biological impact of TE and cytotoxic agent therapy on CCA tissues, CCA xenograft tumours were stained with the proliferation marker Ki67. In iCCA CC‐LP‐1 xenografts, compared with controls, the reduction in tumour cell proliferation by TE, GemCis, and NPT was 43%, 35% and 45%, respectively. The addition of TE to chemotherapy had a synergistic effect on intratumoral proliferation reduction: GemCis+TE (49%) and NPT+TE (63%) (Figure 4A).

In dCCA TFK‐1 xenografts, tumour cell proliferation was reduced by 42%, 39% and 49% by TE, GemCis, and NPT therapy. Again, combination therapy groups, GemCis+TE (62%) and NPT+TE (67%), demonstrated a synergistic effect on the reduction in tumour cell proliferation (Figure 4B).

In the PDX TM00311 study, in line with tumour growth inhibition data, GemCis showed a significantly higher reduction in tumour cell proliferation (62%) compared to NPT (45%). TE's effect on reducing tumour cell proliferation (31%) was less pronounced than that observed in the CC‐LP‐1 or TFK‐1 cell‐derived xenografts. Importantly, the addition of TE to chemotherapy led to a further reduction in tumour cell proliferation: GemCis+TE (86%) and NPT+TE (64%) (Figure 4C).

### 3.5 Effect of Telotristat ethyl and cytotoxic agents on intratumoral serotonin expression

**FIGURE 5:**
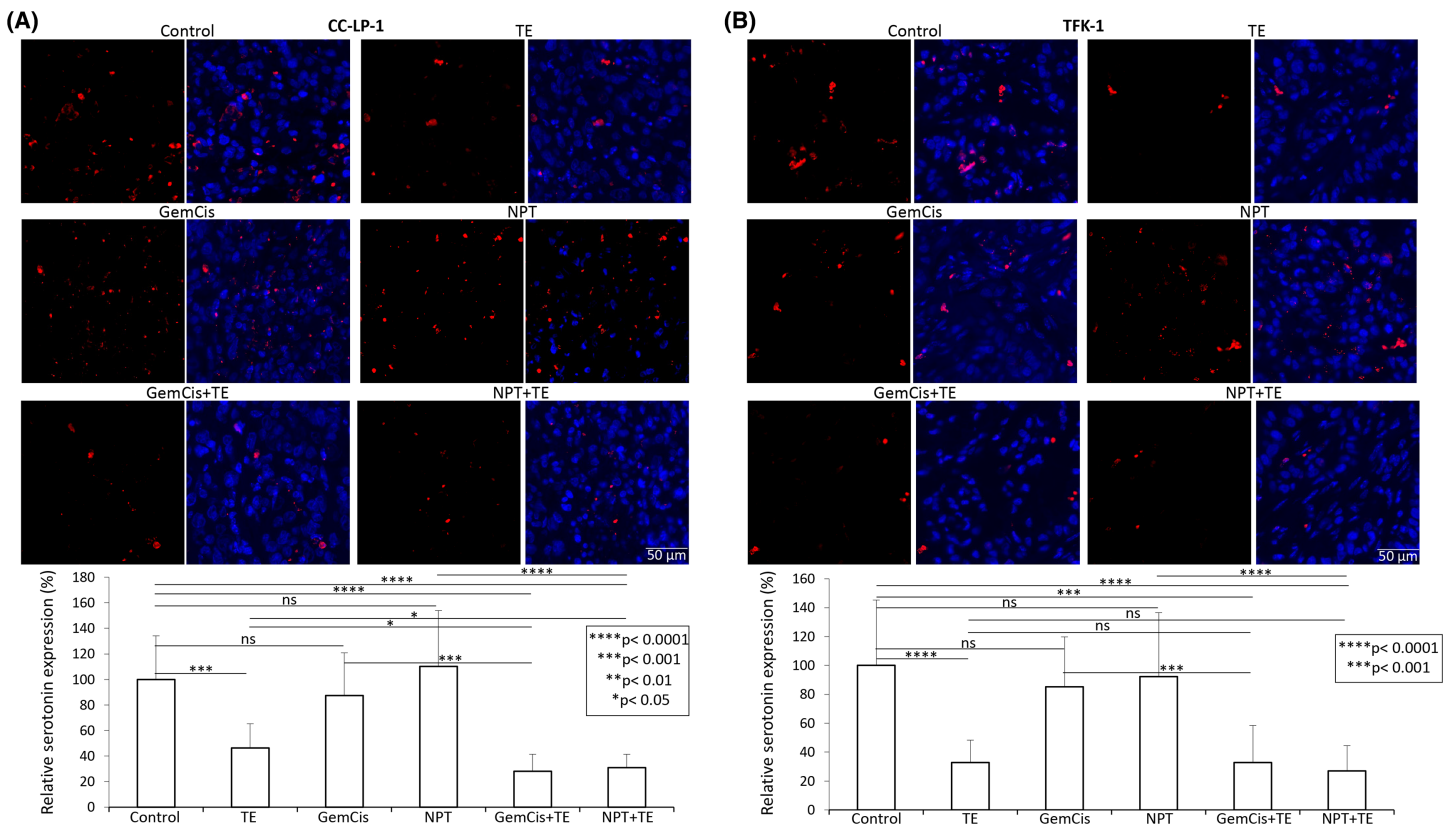
Reduction in serotonin levels by TE, GemCis and NPT treatment in CCA cell‐derived xenografts: Tumour sections from (A) iCCA CC‐LP‐1 and (B) dCCA TFK‐1 subcutaneous xenografts were immunostained with serotonin antibody and photographed under a fluorescent microscope. Serotonin expression was analysed by measuring fluorescence intensity using Image J software. The upper panel depicts images of serotonin staining (left) and merged images of cells stained with serotonin (red) and DAPI (blue) (right) illustrated at 20× magnification. The data are expressed as the mean ± standard deviation. Student's *t*‐test was performed between control and therapy groups, between TE and combination therapy groups, and between chemotherapy and combination therapy groups (*p < 0.05; **p < 0.01; ***p < 0.001; ****p < 0.0001).

The impact of TE and cytotoxic agents on intratumoral serotonin expression was assessed in CCA xenografts. In iCCA CC‐LP‐1 xenografts, TE treatment led to a substantial reduction in serotonin expression, while GemCis and NPT had negligible effects. Notably, the combination therapy groups, GemCis+TE and NPT+TE, also exhibited a significant decrease in serotonin levels (Figure 5A). The percent reduction in serotonin levels for TE, GemCis+TE, and NPT+TE was 54%, 72% and 70%, respectively (Figure 5A).

In dCCA TFK‐1 xenografts, similar to CC‐LP‐1 xenografts, TE treatment resulted in a significant reduction in serotonin levels (67%), whereas GemCis and NPT had no effect. Again, the reduction in serotonin levels after GemCis+TE (67%) and NPT+TE (73%) was not significantly different from TE alone (Figure 5B).

**FIGURE 6:**
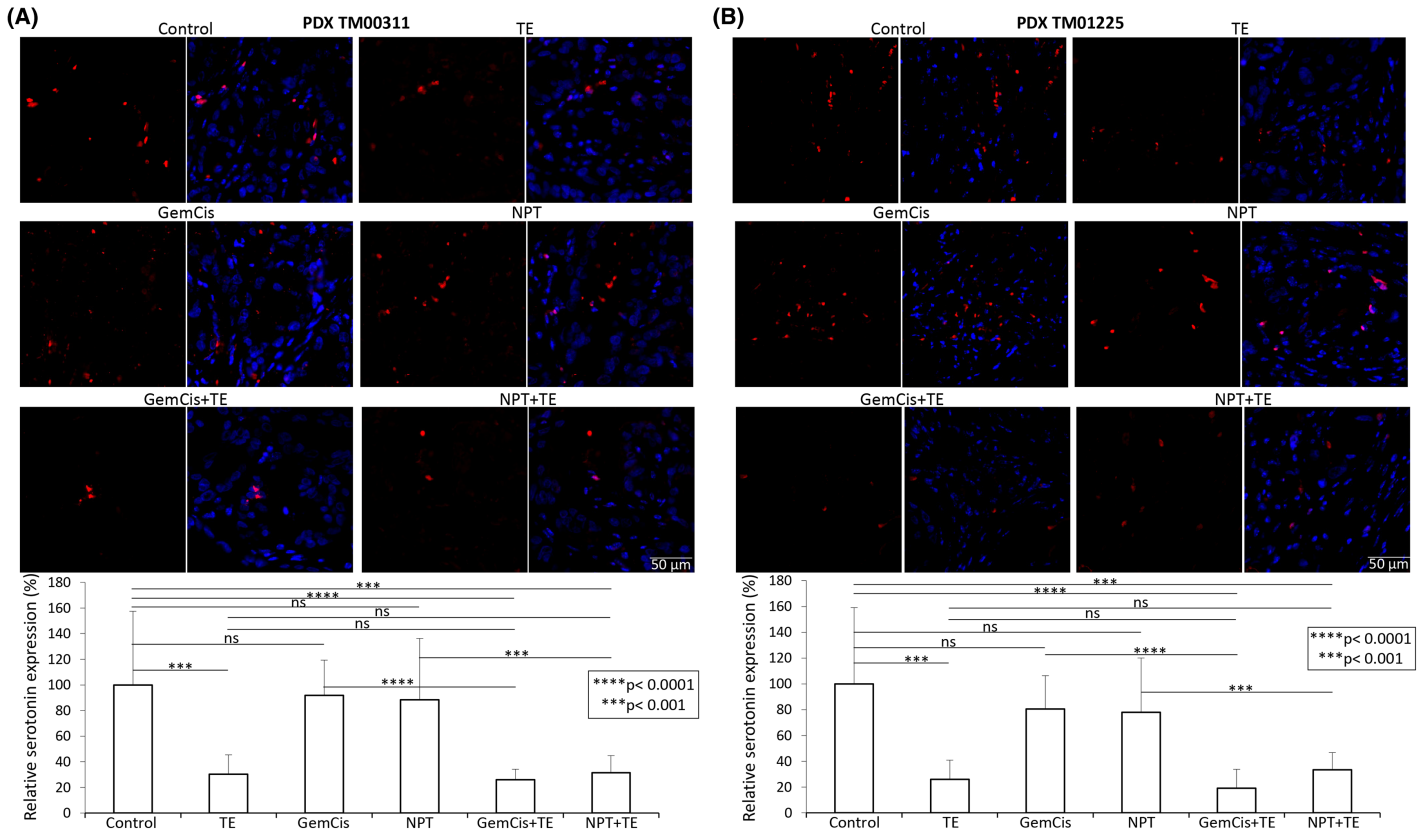
Reduction in serotonin levels by TE, GemCis and NPT treatment in CCA patient‐derived xenografts: Tumour sections from (A) PDX TM00311 and (B) PDX TM01225 subcutaneous xenografts were immunostained with serotonin antibody and photographed under a fluorescent microscope. Serotonin expression was analysed by measuring fluorescence intensity using Image J software. The upper panel depicts images of serotonin staining (left) and merged images of cells stained with serotonin (red) and DAPI (blue) (right) illustrated at 20× magnification. The data are expressed as the mean ± standard deviation. Student's *t*‐test was performed between control and therapy groups, between TE and combination therapy groups, and between chemotherapy and combination therapy groups (***p < 0.001; ****p < 0.0001).

In PDX TM00311 (Figure 6A) and TM01225 (Figure 6B), TE demonstrated a substantial inhibitory effect on serotonin expression in both PDX tumours, resulting in 69.8% and 74.2% inhibition, respectively. The chemotherapy regimens alone did not induce a significant effect on serotonin in either PDX tumours. Furthermore, the combination of TE with chemotherapy did not exhibit a different level of serotonin expression inhibition compared to TE monotherapy in both PDX tumours (Figure 6A,B). These findings highlight the robust inhibitory impact of TE on intratumoral serotonin expression, irrespective of the presence of cytotoxic agents.

### 3.6 Alterations in the expression of marker proteins by telotristat ethyl

**FIGURE 7:**
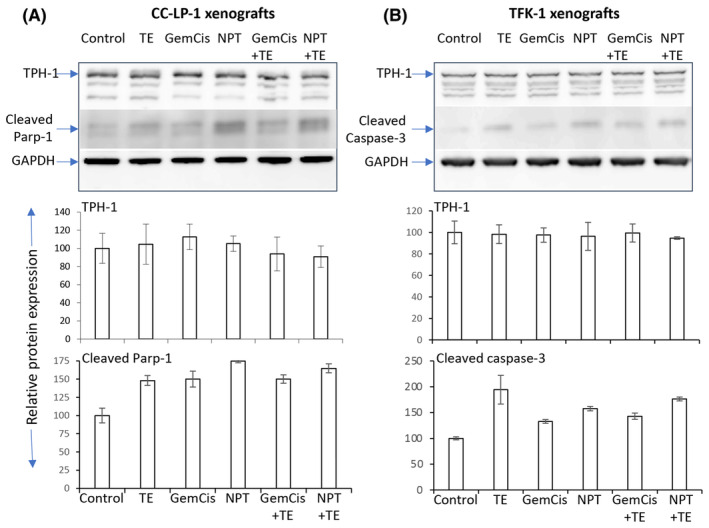
TE effects on marker protein expression in CCA (A) CC‐LP‐1 and (B) TFK‐1 cell‐derived xenografts: Tumour lysates were prepared, and Western blot analyses were performed. The images represent Western blot data of at least three independent experiments with identical outcomes. The intensity of protein bands was quantitated by densitometry and represented in the bar graph after normalizing values with corresponding total protein or GAPDH.

To gain further insights into the molecular mechanisms underlying the efficacy of TE, we conducted Immunoblot analysis of CCA CDX lysates. In both CC‐LP‐1 and TFK‐1 tumour lysates, consistent with the tumour growth inhibition data, TE led to an increase in the expression of apoptosis‐related proteins, cleaved PARP‐1 or cleaved caspase‐3. Neither TE nor cytotoxic regimens had any effect on TPH‐1 levels (Figure 7).

## 4 DISCUSSION

The systemic treatment of CCA presents significant challenges due to the limited clinical efficacy, chemoresistance and associated toxicities of current standard therapies. The substantial heterogeneity of CCAs at the genomic, epigenetic, and molecular levels is a major factor contributing to the diminished effectiveness of these standard treatments. Advancements in the molecular characterization of CCA have led to the development of targeted therapies aimed at improving clinical outcomes for patients. Approximately 50% of CCA patients exhibit druggable mutations, amplifications or fusions, including those involving *FGFR2*, *IDH1/2*, *KRAS*, *BRAF*, *HER2*, *PIK3CA*, and *MET*, presenting opportunities for therapeutic interventions.^30^ The recent FDA approvals of FGFR inhibitors pemigatinib, infigratinib and futibatinib,^31^ as well as the mutant IDH1 inhibitor ivosidenib,^32^ for the treatment of advanced CCA patients underscore the potential of targeted therapies in addressing this aggressive disease. However, it is important to note that these approved targeted therapies are limited to a small subset, primarily of intrahepatic CCAs, making the identification of more common targetable parameters crucial for therapeutic exploration.

Serotonin has emerged as a contributor to tumorigenesis in several human cancers. Promoting cancer cell proliferation, differentiation, migration, metastatic dissemination and angiogenesis.^33^, ^34^ The effects of serotonin on tumour growth are intricate, as its impact appears to be tissue‐specific. Lower doses of serotonin have been associated with vasoconstriction in tumour cells, resulting in tumour growth inhibition. Serum serotonin levels have been identified as prognostic markers in several cancers, highlighting the serotonin pathway as a potential therapeutic target.^33^, ^34^, ^35^ A recent retrospective study analysed tumours from 23 CCA patients treated with GemCis and found that tumour TPH‐1 expression did not predict treatment response. However, a trend toward worse OS was observed in patients with high serum serotonin concentration.^36^ Within the serotonin pathway, serotonin receptors, selective serotonin transporters and the serotonin synthesis pathway represent potential molecular targets for cancer therapy. Nevertheless, the use of serotonin receptor antagonists as anticancer agents in humans is limited due to their side effects, suggesting that inhibiting serotonin biosynthesis holds significant therapeutic potential.^35^ Given the secretion, accumulation and oncogenic impact of serotonin in CCA patients, which corelates with poor prognosis, our study aimed to investigate the antitumor potential of the novel serotonin biosynthesis inhibitor, telotristat ethyl, in preclinical models representing different CCA subtypes.

Metastasis to the peritoneum is the predominant pattern in CCA patients, where GemCis remains the only treatment option, albeit with limited efficacy and a poor prognosis.^37^, ^38^ After evaluating various CCA cell lines, we established an animal survival model for CCA peritoneal dissemination using iCCA CC‐LP‐1 cells. These cells exhibited aggressive metastatic progression and subcutaneous tumour growth, with high expression of BAP‐1, TPH‐1 and serotonin, along with *TP53* mutations. In our study, single‐agent TE did not provide obvious survival benefits, while the addition of TE to GemCis (but not to NPT) led to measurable improvements in chemotherapy‐based survival. In contrast, in subcutaneous xenografts representing all three CCA subtypes (iCCA, pCCA and dCCA), TE monotherapy significantly inhibited tumour growth, and TE addition exhibited a synergistic response when combined with GemCis or NPT. This disparity in TE's efficacy between the two settings may be attributed to differences in the tumour microenvironment, including the presence of dense desmoplastic stroma and metastatic disease at the initiation of treatment. Notably, in both peritoneal dissemination and CDX models, NPT monotherapy exhibited significantly greater antitumor effects compared to GemCis. The advantages of nanoparticle‐based chemotherapy include enhanced transport, permeability, and retention, resulting in increased tissue distribution and tumour penetration, potentially due to its anti‐mitotic and anti‐stromal properties. It is possible that enhanced delivery of TE into the tumour microenvironment may increase its combination effects together with chemotherapeutic agents.

In all three CCA subtypes, TE effectively inhibited tumour growth, although its impact was somewhat reduced in SNU‐1196 pCCA xenografts compared to CC‐LP‐1 iCCA or TFK‐1 dCCA xenografts. This variation aligned with the TPH‐1 levels in these cell lines, with the highest TPH‐1 levels observed in CC‐LP‐1 cells and the lowest in SNU‐1196 cells (Figure S2).

While clinical trial data leading to the approval of the GemCis regimen for treating advanced CCA patients indicated its tolerability, several studies have reported significant toxicity associated with this regimen.^39^, ^40^ In our study, using gemcitabine and cisplatin at 50% of their maximum tolerated dose (MTD) in GemCis groups resulted in therapy‐related weight loss, necessitating the adjustment of chemotherapy doses to 25% of their MTD, which was then tolerated well. In contrast, TE and NPT were well tolerated when administered alone or in combination. Importantly, we observed that mouse weight loss caused by GemCis+TE therapy was less pronounced than that induced by GemCis therapy alone in all xenograft studies, suggesting TE's potential to mitigate chemotherapy‐related weight loss. These findings align with a clinical study in which up to 33% of patients treated with TE experienced significant, dose‐dependent weight gain.^41^

In contrast to CDX studies, GemCis demonstrated significantly higher antitumor efficacy in treatment‐naïve PDX TM00311 compared to NPT. Another PDX model, TM01225, for which the treatment status was unknown, exhibited a similar level of GemCis efficacy, suggesting that this PDX may also have been treatment‐naïve. The reduced sensitivity of GemCis in CDX studies compared to NPT raises the possibility that the CCA cells used to establish CDX models may have originated from GemCis‐treated tumours, which could have developed resistance to this regimen.

Biomarkers such as high tumour mutational burden (TMB‐H), high microsatellite instability (MSI‐H), DNA mismatch repair deficiency (MMRd), and the expression of immune checkpoint proteins have been associated with sensitivity to immune checkpoint inhibitor (ICI) therapy in several cancers.^42^ ICI therapy for CCA patients carries promise, given some of the tumours' immunologic features, including the expression of immune checkpoint molecules, such as PD‐L1 and cytokine T‐lymphocyte‐associated protein 4 (CTLA‐4) within the tumour microenvironment.^43^, ^44^, ^45^ The recent approval of durvalumab in combination with GemCis for the treatment of advanced CCA has underscored the potential of ICI therapy in this disease.^12^ Considering that attenuation of peripheral serotonin inhibits tumour growth and enhances ICI therapy by promoting CD8+ T cell accumulation in some tumours,^46^ our study opens avenues for evaluating TE in combination with ICI therapy and nanoparticle‐based chemotherapy. Recently, a phase 2 study of the TE combination with GemCis in unresectable, previously untreated, advanced CCA patients demonstrated encouraging OS of 17.67 months; however, the study was terminated as it did not meet the pre‐specified criteria for a 6‐month PFS (NCT03790111, clinicaltrials.gov).

In recent years, targeted therapies have revolutionized the treatment of CCA patients by selectively blocking potential pathways responsible for CCA growth and metastasis, such as the FGFR and IDH1/2 signalling pathways. In light of the oncogenic potential of serotonin and the dysregulated serotonin metabolic pathway in CCA, particularly the increased expression of TPH1 resulting in elevated serotonin accumulation and secretion, our study emphasizes the therapeutic potential of TE. This investigation has unequivocally demonstrated the significant antitumor efficacy of TE across a spectrum of CCA preclinical models, highlighting the potential for combination therapies involving TE, particularly for patients exhibiting serotonin overexpression. Such approaches hold the promise of improving clinical outcomes for advanced CCA patients.

## AUTHOR CONTRIBUTIONS

**Niranjan Awasthi:** Conceptualization (equal); data curation (lead); formal analysis (lead); funding acquisition (equal); investigation (lead); methodology (equal); resources (equal); supervision (equal); validation (equal); visualization (equal); writing – original draft (lead); writing – review and editing (lead). **Lily Darman:** Data curation (supporting); formal analysis (supporting); methodology (supporting). **Margaret A. Schwarz:** Data curation (equal); formal analysis (equal); methodology (equal). **Roderich E. Schwarz:** Conceptualization (equal); funding acquisition (equal); supervision (equal); writing – review and editing (equal).

## FUNDING INFORMATION

This research work was financially supported by Lexicon Pharmaceuticals, TerSera Therapeutics, and Indiana University School of Medicine funds.

## CONFLICT OF INTEREST STATEMENT

The authors N. Awasthi and R.E. Schwarz received funding support from Lexicon Pharmaceuticals and TerSera Therapeutics to conduct this research. The authors Lily Darman and M.A. Schwarz declare no conflict of interest.

## Supporting information


**Figure S1:** Chemical structure of telotristat ethyl.

**Figure S2:** Expression of tryptophan hydrogenase 1 (TPH1) in CCA cell lines. Whole cell lysates were prepared from subconfluent cultures of the cell lines then TPH1 expression was determined via Western blot analysis.

**Table S1:** Characteristics of the human cholangiocarcinoma cancer cell lines used in the study. iCCA, intrahepatic cholangiocarcinoma; dCCA, distal cholangiocarcinoma; pCCA, perihilar cholangiocarcinoma; m, mutation; a, amplification.

**Table S2:** Characteristics of cholangiocarcinoma PDX models.

## Data Availability

The data supporting the findings of this study are included within the article and its supplementary materials. Additional raw data can be obtained from the corresponding author upon request.
