# Supplementary figures and images for: Telotristat ethyl, a tryptophan hydroxylase inhibitor, enhances antitumor efficacy of standard chemotherapy in preclinical cholangiocarcinoma models
Source: J Cell Mol Med. 2024 Sep 2;28(17):e18585. doi: 10.1111/jcmm.18585 (PMC11369204; doi:10.1111/jcmm.18585)

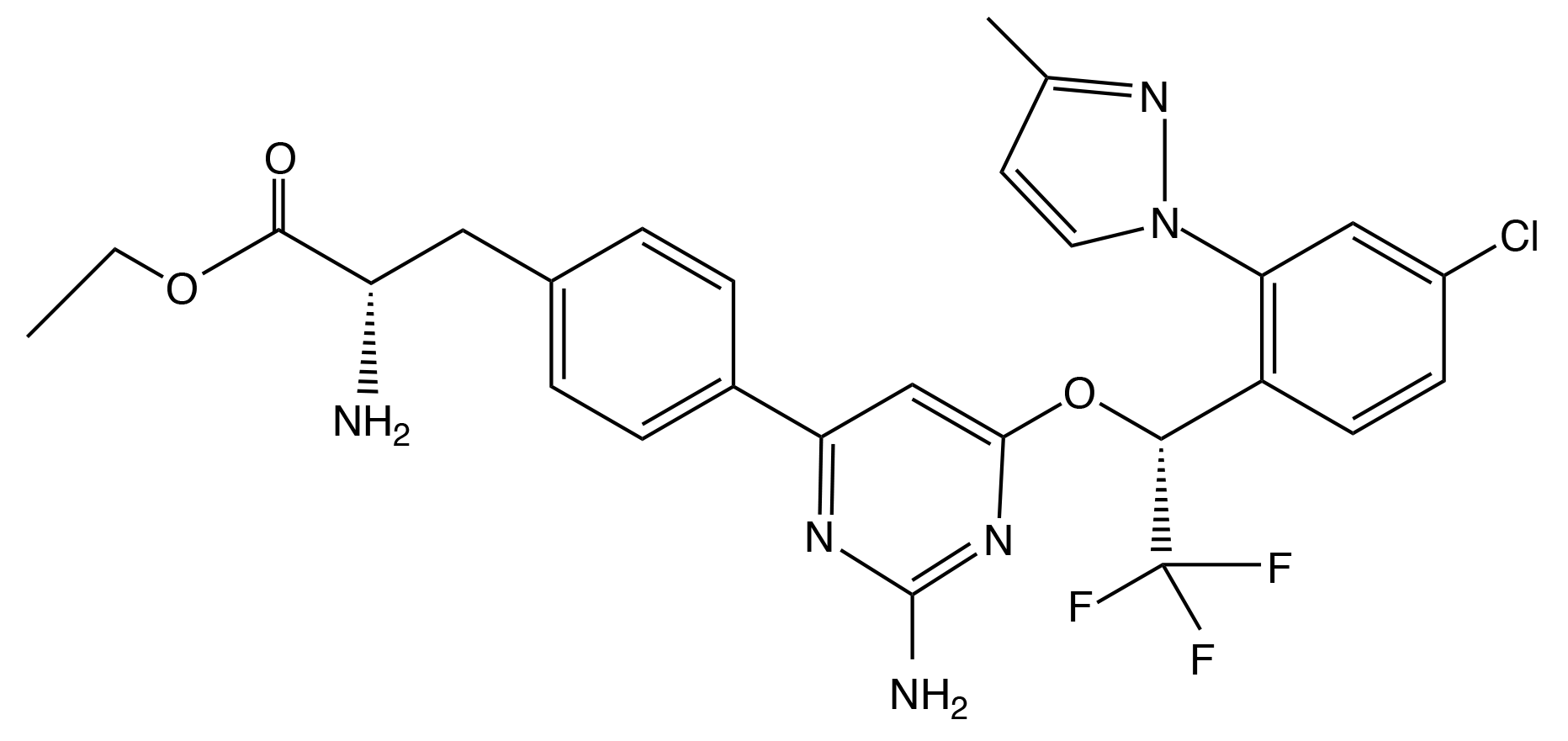

Supplement: Supplementary file 1 — Figure S1: Chemical structure of telotristat ethyl. [file JCMM-28-e18585-s003.tif]

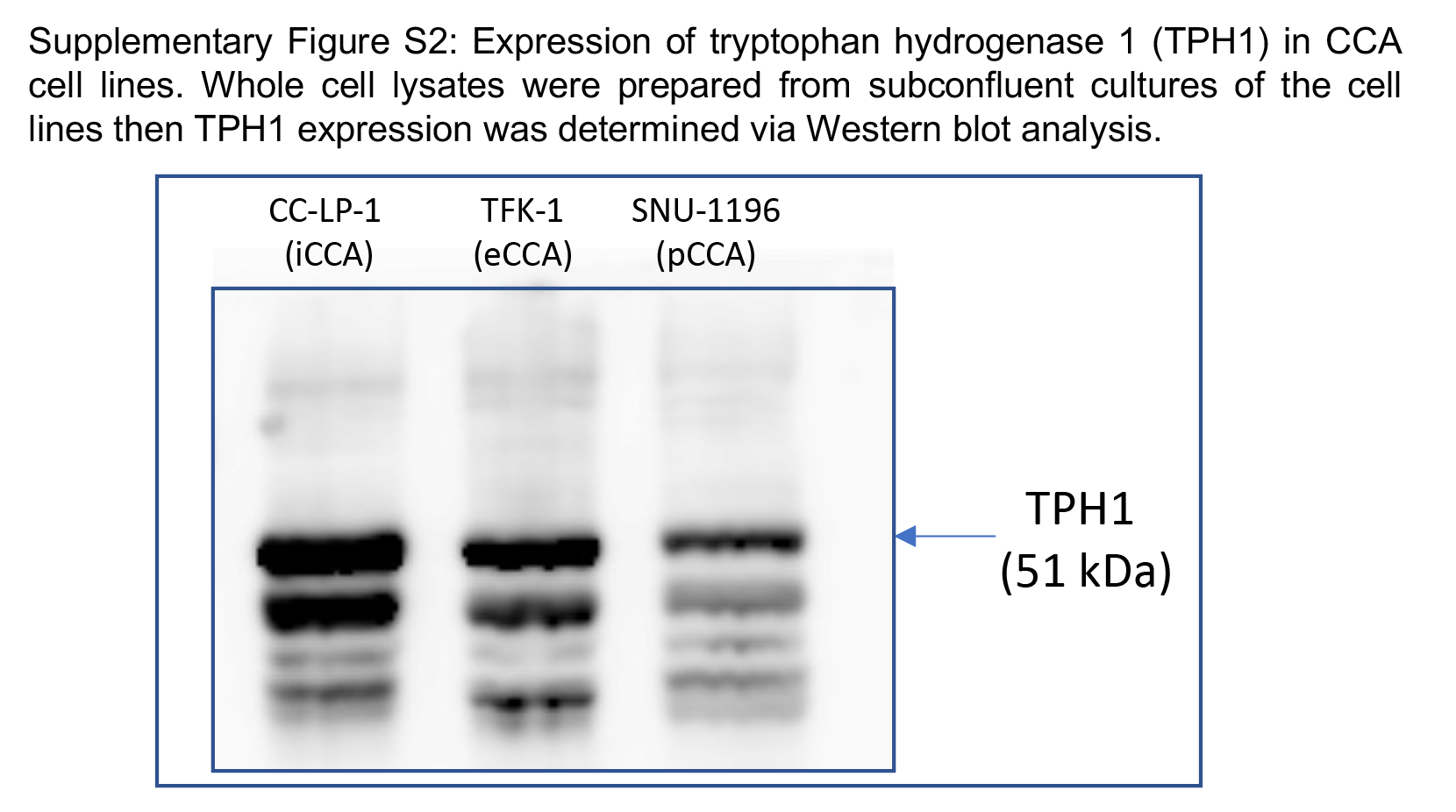

Supplement: Supplementary file 2 — Figure S2: Expression of tryptophan hydrogenase 1 (TPH1) in CCA cell lines. Whole cell lysates were prepared from subconfluent cultures of the cell lines then TPH1 expression was determined via Western blot analysis. [file JCMM-28-e18585-s004.tif]

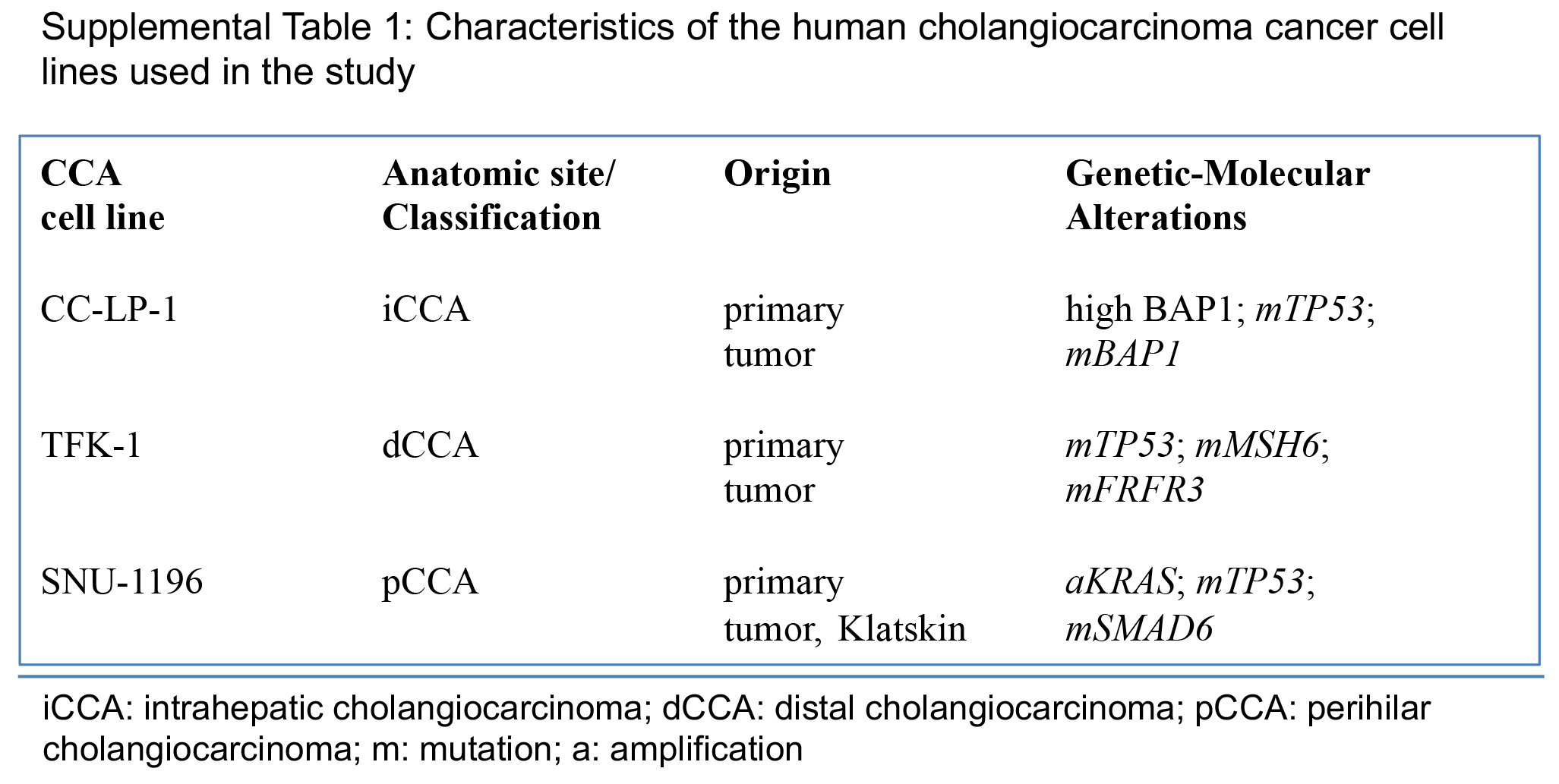

Supplement: Supplementary file 3 — Table S1: Characteristics of the human cholangiocarcinoma cancer cell lines used in the study. iCCA, intrahepatic cholangiocarcinoma; dCCA, distal cholangiocarcinoma; pCCA, perihilar cholangiocarcinoma; m, mutation; a, amplification. [file JCMM-28-e18585-s002.tif]

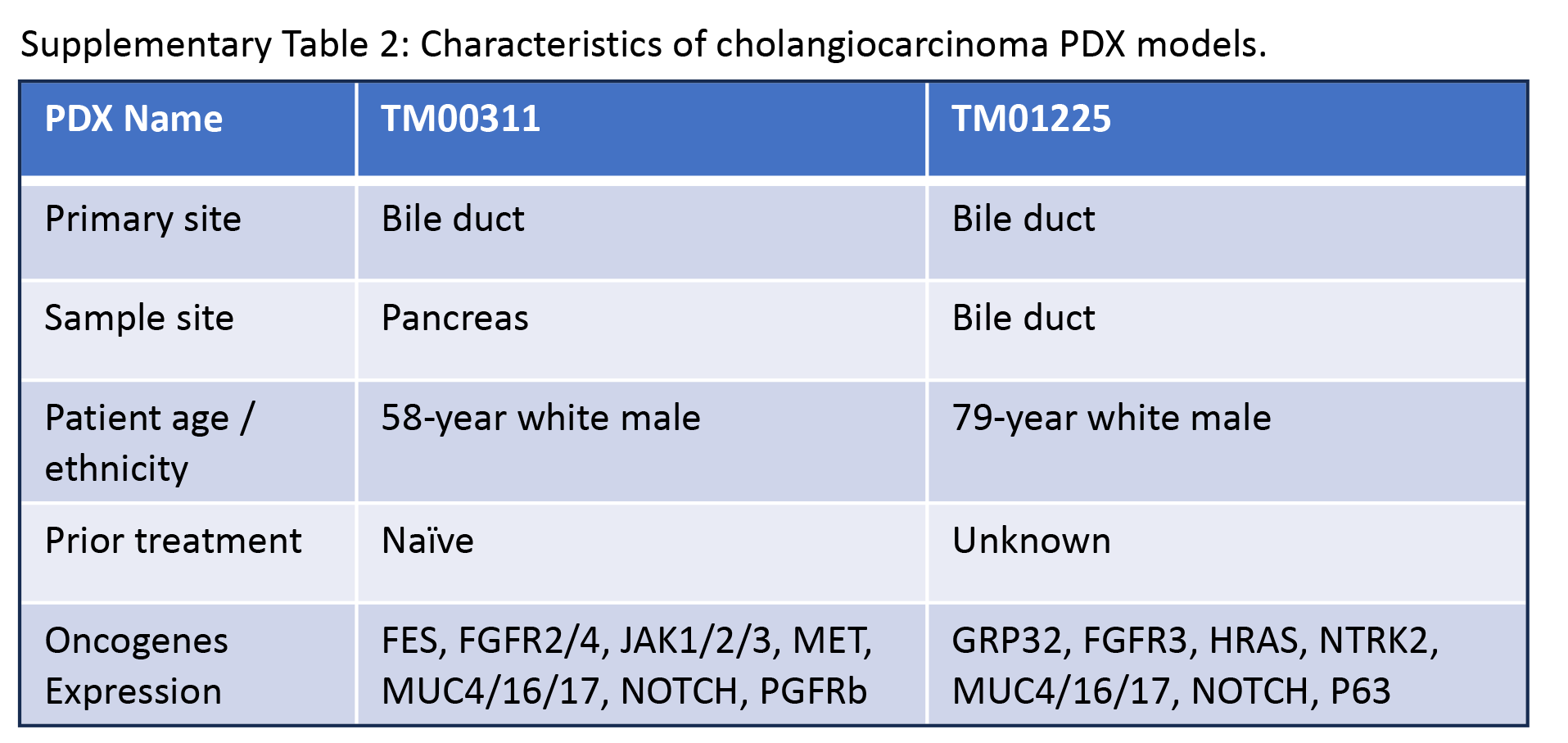

Supplement: Supplementary file 4 — Table S2: Characteristics of cholangiocarcinoma PDX models. [file JCMM-28-e18585-s001.tif]
